# Severe tracheobronchial stenosis and bronchiectasis complicating ulcerative colitis

**DOI:** 10.1002/rcr2.45

**Published:** 2014-01-21

**Authors:** Toshio Suzuki, Kenji Tsushima, Yuichi Sakairi, Shigetoshi Yoshida, Ichiro Yoshino, Koichiro Tatsumi

**Affiliations:** 1 Department of Respirology, Graduate School of Medicine, Chiba UniversityChiba, Japan; 2 Department of General Thoracic Surgery, Graduate School of Medicine, Chiba UniversityChiba, Japan

**Keywords:** Bronchiectasis, main bronchial stenosis, ulcerative colitis, YAG laser

## Abstract

A 37-year-old woman with a 20-year history of ulcerative colitis (UC) was admitted with complaints of cough and increasing sputum production. Chest computed tomography showed severe stenosis of the left main bronchus and bronchiectasis of the left lower lobe. Biopsy specimens from the area of bronchial stenosis showed chronic inflammation with lymphocyte infiltration, and we diagnosed respiratory involvement of UC. The bronchial stenosis was successfully treated with yttrium aluminum garnet (YAG) laser. UC is a systemic illness with occasional extraintestinal manifestations, but upper airway involvement is rare, and to our knowledge, this is the first published report of UC complicated with bronchopulmonary lesions with successful YAG laser treatment of the main bronchial stenosis.

## Introduction

Clinically significant lung with ulcerative colitis (UC) involvement is rare and may take the form of chronic bronchitis, bronchiectasis, granulomatous interstitial pneumonitis, bronchiolitis obliterans, fibrosis, alveolitis, or pleuritis [1]. Upper airway involvement in UC is even more unusual and consists of mucosal inflammation [2]. We report a case of UC with severe main bronchial stenosis and presented successful treatment of a severe main bronchial stenosis by endoscopic destruction through the action of yttrium aluminum garnet (YAG) laser.

## Case Report

**Figure 1:**
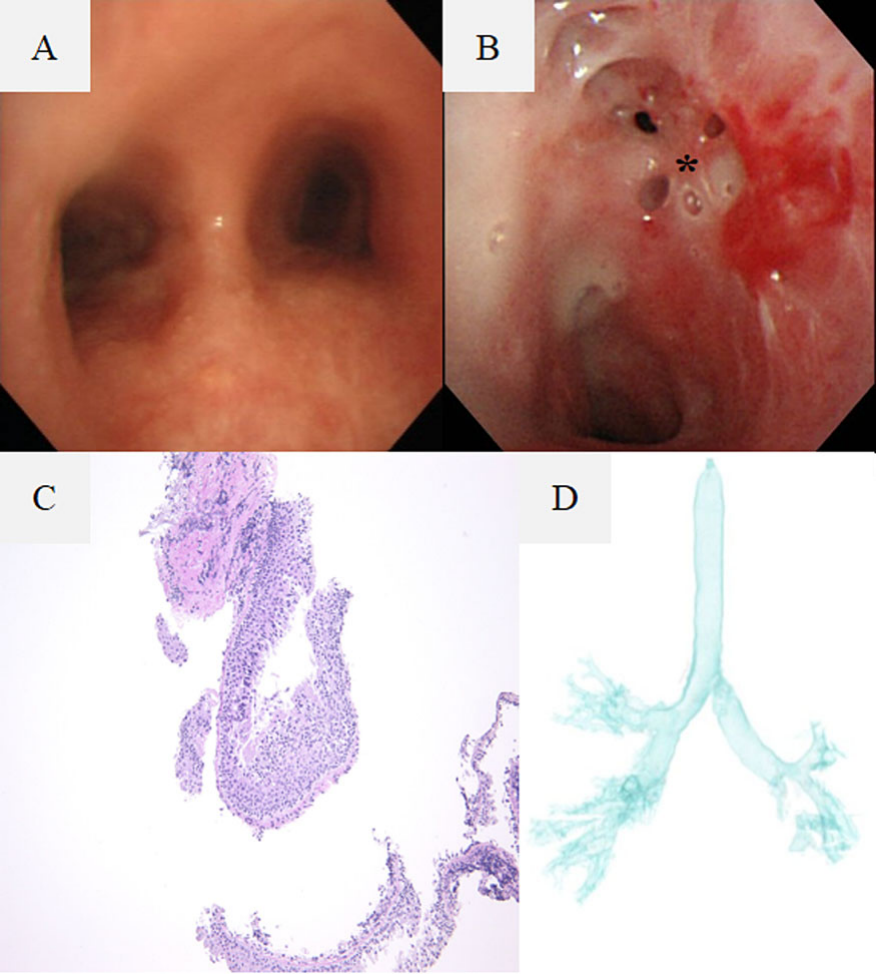
(A) Bronchoscopic findings showed edematous change in the trachea and main bronchi in the year 2000. (B) Bronchoscopic findings showed severe stenosis of the left main bronchus in 2012. There was a membranous web partially obstructing the left main bronchus. (C) The stenosed area showed lymphocytic infiltration under the basement membrane (hematoxylin and eosin stain; original magnification, 40×). (D) A three-dimensional chest computed tomography on the most recent admission showed severe membranous stenosis of the left main bronchus.

A 37-year-old woman who had been diagnosed with UC in 1993 presented with persistent cough. In 1996, the patient had developed a persistent cough with purulent sputum, but she remained without any abnormalities on chest X-ray, although chest computed tomography (CT) revealed bronchiectasis in the left lower lobe. Repeated sputum cultures proved to be negative for bacteria, acid-fast bacilli, and fungi. The patient's respiratory symptoms persisted despite the cessation of oral prednisolone, and bronchoscopy in the year 2000 revealed edematous change and irregular mucosa in the trachea and main bronchi (Fig. 1A). Treatment with low-dose oral erythromycin was initiated. The patient presented with increasing sputum production in 2010. The chest X-ray revealed severe stenosis of the left main bronchus. A three-dimentional chest CT confirmed the stenosis of the left main bronchus (Fig. 1D) and showed deformity of the left lower lung field. There were no particular abnormalities among the laboratory findings (Table 1), and the interferon-γ releasing assay and serum antineutrophil cytoplasmic antibodies (ANCA) were negative. Sputum culture revealed *Pseudomonas aeruginosa*. A bronchoscopy showed extensive severe stenosis of the left main bronchus (Fig. 1B), and several biopsy specimens obtained from the stenosed areas showed submucosal lymphocyte infiltration (Fig. 1C). The stenosis was treated by endoscopic destruction through the action of YAG laser. After treatment, the vital capacity and forced expiratory volume in 1 sec increased from 2420 to 2560 mL and from 1600 to 2080 mL, respectively. There was no restenosis at the 6-month follow-up, and the patient underwent resection of the left lower lung abscess. *P. aeruginosa* was also cultured from the resected lung, but there was no evidence of malignancy, granuloma, or amyloid deposit. The proximal bronchial mucosa in the resected lung was smooth and without lymphocyte infiltration pathohistologically.

**Table 1:** Laboratory data on admission

Hematology			Biochemistry			Serology		
WBC	10,700	/μL	TP	7.3	g/dL	CRP	0.3	mg/dL
Neutrophil	76.6	%	Alb	4.2	g/dL	CYFRA	0.6	U/mL
Lymphocyte	17.1	%	AST	26	IU/L	CA19-9	16.4	U/mL
Monocyte	4.7	%	ALT	33	IU/L	ProGRP	35.9	pg/mL
Eosinophil	1.1	%	LDH	171	IU/L	CEA	0.7	pg/mL
Basophil	0.5	%	ALP	258	IU/L	β-D glucan	<3.14	pg/mL
RBC	440 × 10^4^	/μL	BUN	10	mg/dL	PR3-ANCA	<20	EU
Hb	13.0	g/dL	Cre	0.67	mg/dL			
Hct	38.5	%	Na	140	mEq/L			
Plt	295,000	/μL	K	4.1	mEq/L			
			Cl	107	mEq/L			

Alb, albumin; ALP, alkaline phosphatase; ALT, alanine aminotransferase; AST, aspartate aminotransferase; BUN, blood urea nitrogen; CA19-9, carbohydrate antigen 19-9; CEA, carcinoembryonic antigen; Cl, chlorine; Cre, creatinine; CRP, C-reactive protein; CYFRA, cytokeratin fragment; Hb, hemoglobin; Hct, hematocrit; K, kalium; LDH, lactate dehydrogenase; Na, natrium; Plt, platelet; ProGRP, pro-gastrin releasing peptide; PR3-ANCA, proteinase-3 antineutrophil cytoplasmic antibody; RBC, red blood cell; TP, total protein; WBC, white blood cell.

## Discussion

The present case is the first reported case to demonstrate severe involvement of both upper and lower airway in UC. There are several infectious and noninfectious inflammatory processes, neoplasms, and exogenous factors that can lead to main bronchial stenosis. In the present case, repeated sputum cultures prior to the most recent admission had revealed no evidence of bacterial infection (including *Mycobacterium* species). Although the sputum culture on the recent admission revealed *P. aeruginosa*, which was also later cultured from the resected left lower lung abscess, the stenosed area of the main bronchus was apart from the left lower lung field, and the other areas of bronchial mucosa in the resected lung specimen were normal. Furthermore, there have been no previous reports of main bronchial stenosis induced by chronic *P. aeruginosa* infection. We believe that the administration of low-dose oral erythromycin for chronic bronchitis and bronchiectasis may have led to *P. aeruginosa* adherence in the lung. The histological examination of the area of bronchial stenosis showing chronic inflammation with lymphocytes ruled out bronchial amyloidosis, relapsing polychondritis or granulomatous disease (i.e. sarcoidosis, tuberculosis, or Wegener's granulomatosis), and the laboratory findings showed a normal range of ANCA and normal urinary sediment. The patient did not have any oral ulcers or purulent nasal discharge, which are clinical findings that may have supported a diagnosis of Wegener's granulomatosis.

Severe upper airway stenosis with UC has previously been described in only three cases [2, 3]. The pathophysiological mechanism of UC airway involvement remains unclear. Generally, the inflammatory features of UC include increased cellularity of the lamina propria, basal plasmacytosis, basal lymphoid aggregates, and lamina propria eosinophilia. No pathological features specific to airway involvement of UC [2, 3] have been identified. Rickli et al. reported a UC patient with severe inflammatory upper airway stenosis with mucosal biopsy specimens showing extensive lymphoplasmacytic inflammation [3]. In our case, the obstructive mucosa contained a lymphocytic infiltration. Camus et al. have suggested that the origin of both colon and bronchi in primary gut may predispose UC patients to the possibility of bronchial involvement [4], and it has been reported that mesenteric vasculitis is thought to be related to inflammatory bowel diseases (IBDs), which also suggests a potential for UC, as a disease related to systemic vasculitis to be complicated with involvement of both colon and lungs [5].

The treatment of IBD-related respiratory disease depends on the disease pattern. Neither coloproctectomy nor classic nonsteroidal IBD-modifying drugs, including immunosuppressants, have demonstrated any effect in controlling respiratory manifestations of IBD, except in rare instances [1]. In fact, colonic surgery may aggravate prior airway disease [1]. Although most respiratory complications associated with IBD respond to steroids, it is more difficult to treat for airway disease than for other forms [1]. In the present case, the patient's upper airway disease might have progressed after cessation of oral prednisolone. Camus et al. have reported that treatment with steroids is effective in IBD-related large airways disease, and that inhaled steroids seem far more effective and are better tolerated than oral steroids [1].

In summary, we have reported a case of UC with severe bronchial stenosis and bronchiectasis in the left lower lobe.
